# Metformin-induced lactic acidosis: a case series

**DOI:** 10.1186/1752-1947-1-126

**Published:** 2007-10-31

**Authors:** Joana Silvestre, Susana Carvalho, Vitor Mendes, Luis Coelho, Camila Tapadinhas, Pedro Ferreira, Pedro Povoa, Fatima Ceia

**Affiliations:** 1 Department of Medicine III, Unidade de Cuidados Intensivos Médicos, Serviço de Medicina III, Hospital São Francisco Xavier, Portugal; 2 Department of Anaesthesiology, Unidade de Cuidados Intensivos Médicos, Serviço de Medicina III, Hospital São Francisco Xavier, Portugal

## Abstract

**Introduction:**

Unlike other agents used in the treatment of type 2 diabetes mellitus, metformin has been shown to reduce mortality in obese patients. It is therefore being increasingly used in higher doses. The major concern of many physicians is a possible risk of lactic acidosis. The reported frequency of metformin related lactic acidosis is 0.05 per 1000 patient-years; some authors advocate that this rate is equal in those patients not taking metformin.

**Case presentation:**

We present two case reports of metformin-associated lactic acidosis. The first case is a 77 year old female with a past medical history of hypertension and type 2 diabetes mellitus who had recently been prescribed metformin (3 g/day), perindopril and acetylsalicylic acid. She was admitted to the emergency department two weeks later with abdominal pain and psychomotor agitation. Physical examination revealed only signs of poor perfusion. Laboratory evaluation revealed hyperkalemia, elevated creatinine and blood urea nitrogen and mild leukocytosis. Arterial blood gases showed severe lactic acidemia. She was admitted to the intensive care unit. Vasopressor and ventilatory support was initiated and continuous venovenous hemodiafiltration was instituted. Twenty-four hours later, full clinical recovery was observed, with return to a normal serum lactate level. The patient was discharged from the intensive care unit on the sixth day. The second patient is a 69 year old male with a past medical history of hypertension, type 2 diabetes mellitus and ischemic heart disease who was on metformin (4 g/day), glycazide, acetylsalicylic acid and isosorbide dinitrate. He was admitted to the emergency department in shock with extreme bradycardia. Initial evaluation revealed severe lactic acidosis and elevated creatinine and urea. The patient was admitted to the Intensive Care Unit and commenced on continuous venovenous hemodiafiltration in addition to other supportive measures. A progressive recovery was observed and he was discharged from the intensive care unit on the seventh day.

**Conclusion:**

We present two case reports of severe lactic acidosis most probably associated with high doses of metformin in patients with no known contraindications for metformin prescription. In both patients no other condition was identified to cause such severe lactic acidosis. Although controversial, lactic acidosis should be considered in patients taking metformin.

## Introduction

Metformin, a dimethylbiguanide, is an oral antihyperglycemic drug used to treat type 2 diabetes mellitus. It was approved in the United States by the Food and Drug Administration (FDA) only recently – in 1995.

The results of the United Kingdom Prospective Diabetes Study (UKPDS) have provided good evidence of the benefits of metformin on the long-term incidence of diabetic complications in overweight patients [1].

Biguanides decrease gluconeogenesis from alanine, pyruvate, and lactate. The accumulation of lactic acid may increase under several circumstances[2]. The occurrence of metformin induced lactic acidosis is exceptional when the drug is used with caution.

Lactic acidosis is rare, but serious, with a mortality up to 50% [3]. It is a type of high anion gap metabolic acidosis and is associated with various pathological processes. It has also been used as a prognostic index of mortality [4].

Metformin can be associated with lactic acidosis in patients with other clinical conditions that can themselves cause hyperlactacidemia, namely heart failure, hypoxia and sepsis. The overall incidence of this complication is 0.05 cases per 1,000 patient-years. Almost all of the reported cases occurred in patients who had risk factors for lactic acidosis [5]. But it is impossible to determine to what extent, if any, metformin may have contribute to the development of lactic acidosis in any individual case [6].

Salpeter et al [7] in a meta-analysis of 194 studies found no difference in the incidence of lactic acidosis between diabetics taking and not taking metformin.

We report the cases of two older adults admitted to our institution with severe lactic acidosis in the setting of type 2 diabetes treatment with metformin.

## Case presentation

### Patient 1

A 77-year-old Caucasian female was admitted to the emergency department after two weeks of increasing abdominal pain associated with vomiting. Two days before admission, she developed psychomotor agitation. She had a past medical history of type 2 diabetes, arterial hypertension and cerebrovascular disease. She had had a stroke one month before with full recovery; at that time her creatinine was normal and she had been discharged from hospital with the following medications: metformin 3 g daily, perindopril 8 mg daily, and simvastatin 20 mg daily.

On admission examination revealed a Glasgow Coma Scale score of 12/15 (E_4_V_3_M_5_), blood pressure 136/87 mmHg, pulse 100 beats per minute, respiratory rate 20 breaths per minute and core body temperature 36.6°C. Despite being eupnoeic with oxygen saturation measured by pulse oximetry was 97% on room air, she presented with signs of poor perfusion.

Initial investigations revealed a creatinine of 6 mg/dL, sodium 142 mEq/L, potassium 4.7 mEq/L, chloride 103 mEq/L, glucose 216 mg/dL and C-reactive protein 3.14 mg/dl. Complete blood count (CBC) count showed 22.4 × 10^9^/L white blood cells, with haemoglobin of 13.8 g/dL, and platelet count of 365 × 10^9^/L. Arterial blood gas showed severe lactic acidosis (pH 6.87, PaCO_2 _8.2 mmHg, PaO_2 _146 mmHg, HCO_3_^- ^1.4 mEq/L, blood lactate 16 mmol/L). Chest X-rays and ECG were normal at the time of her admission. Serum toxicological results, namely benzodiazepines, tricyclic antidepressants, opiates and barbiturates, were negative.

The patient was admitted to the intensive care unit (ICU) with the diagnosis of metformin related lactic acidosis. Continuous venovenous haemodialysis (CVVHD) was initiated, with 2 L/h of dyalisate flow and 35 ml/kg/h of hemofiltration using the solutions from Fresenius HF BIC, with 2 and 4 mEq/L of potassium as needed, using a high-flux dyalizer membrane (ultraflux AV 600s). Elective endotracheal intubation and mechanical ventilation was performed.

Four hours after the initiation of CVVHD significant improvement of acid-base status was observed and blood lactate level had halved (table 1). On the third day the patient was successfully weaned from the ventilator. On the 5th day a primary methicillin resistant Staphylococcus aureus bloodstream infection was diagnosed and the patient was started on vancomycin. The patient was discharged to the nephrology department ward on the seventh day.

**Table 1:** Patient 1 – Arterial blood gas results at admission, 4 h and 12 h after initiation of continuous venovenous hemodiafiltration

	0 h	4 h	12 h
**pH**	6.8	7.434	7.4
**PaCO_2 _(mmHg)**	8.2	15	22.9
**PaO_2 _(mmHg)**	146	125	108.4
**SaO_2 _(%)**	97.10	99.5	98.9
**HCO_3_^- ^(mEq/L)**	1.4	10.1	14.1
**Lactate (mmol/L)**	16	7.3	5

Full recovery of renal function was observed after 30 days and the patient was discharged from hospital on the 60th day medicated with insulin and glycazide.

### Patient 2

A 69 year old male was admitted to the emergency department with confusion due to altered mental status. His past medical history included type 2 diabetes, stable angina and hypertension. There was no previous history of hospitalisations. His usual medications included metformin (4 g/day), isosorbide dinitrate (60 mg/day), glycazide (60 mg/day) and acetylsalicylic acid (150 mg/day).

On admission, the patient had extreme bradycardia with pulse rate 30 beats per minute, respiratory rate 28 breaths per minute, core body temperature 36.0°C and the blood pressure was immeasurable using the cuff method. There were no other significant findings on the physical examination. ECG showed complete atrio-ventricular block and the patient was immediately connected to an external pacemaker with significant haemodynamic improvement, blood pressure rising to 127/76 mmHg.

Initial investigations showed creatinine 2.2 mg/dL, sodium 135 mEq/L, potassium 3.4 mEq/L and glucose 436 mg/dL. Arterial blood gas showed severe lactic acidosis (pH 6.7, PaCO_2 _32.4 mmHg, PaO_2 _68.2 mmHg, HCO_3_^- ^5.0 mEq/L, and lactates 18 mmol/L). Hemogram showed a normocytic, normochromic anaemia (haemoglobin 8 g/dL) and mild thrombocytopenia (platelets 130 × 10^9^/L). Microscopic examination of the urine sediment showed the presence of glycosuria. Chest X-rays were normal. Serum toxicological results, namely for benzodiazepines, tricyclic antidepressants, opiates and barbiturates, were negative.

The patient started treatment with fluids, bicarbonate and insulin infusion since the admission diagnosis was ketoacidosis. During the procedure to implant a temporary pacemaker the patient suffered a respiratory-cardiac arrest followed by shock. He was then transferred to the ICU where he received vasopressor support with the diagnosis of metformin related lactic acidosis. Accordingly, CVVHD was initiated with 2 L/h of dyalisate flow and 35 ml/kg/h of hemofiltration using the solutions from Fresenius HF BIC, with 2 and 4 mEq/L of potassium as needed, using a high-flux dyalizer membrane (ultraflux AV 600s).

The patient stabilized after 4 hours of CVVHD with an improvement of acid-base status and a decreased lactate level (table 2). At the 12^th ^hour of ICU stay the patient no longer needed vasopressor support and he recovered stable sinus rhythm and, as a result, the pacemaker was removed. On the third day CVVHD was stopped with full recovery of renal function. On the same day, an early-onset ventilator associated pneumonia was diagnosed and the patient was put on empiric broad-spectrum antibiotic with piperacilin/tazobactam. Blood cultures, bronchial secretions and bronchoalveolar lavage were negative. The patient improved and was successfully weaned from the ventilator on the fifth day and was discharged from hospital on the seventh day.

**Table 2:** Patient 2 – Arterial blood gas results at admission, 4 h and 12 h after initiation of continuous venovenous hemodiafiltration

	0 h	4 h	12 h
**pH**	6.7	7.34	7.43
**PaCO_2 _(mmHg)**	32.4	23.1	29.8
**PaO_2 _(mmHg)**	68.2	62	101.4
**SaO_2 _(%)**	77.1	93.1	97.2
**HCO_3_^- ^(mEq/L)**	5	12	15
**Lactate (mmol/L)**	18	9.8	3.4

## Discussion

There is controversy regarding the association between metformin use and lactic acidosis [6]. The independent effect of metformin on the development of lactic acidosis is unknown. However, metformin-associated lactic acidosis is recognized as a potential lethal condition that can occur in patients with contraindications to the drug, such as renal failure, sepsis, hypoxaemia, and alcoholism [5,8].

In our two case reports, no other aetiology for such severe lactic acidosis was found apart from metformin, despite metformin plasma concentration not having been measured since its determination was not available in Portugal at the time. Both patients were type 2 diabetics without risk factors for developing lactic acidosis such as renal failure. We were also unable to find any known precipitating factor that could contribute to such severe lactic acidosis, namely signs of dehydratation or the presence of toxic drugs or infection. The only common factor in both patients were high doses of metformin, in particular in the second patient since the prescribed metformin daily dose was superior to the FDA recommendations, and advanced age, both patients being older than 70 years. Another unexpected and not described finding was the appearance of acute renal failure in patients without previous renal dysfunction. We question whether high doses of metformin in older patients could be responsible for acute renal failure with subsequent lactic acidosis due to drug accumulation, since metformin is excreted by the kidneys without being metabolized. Some studies have shown a positive correlation between serum creatinine and plasma metformin as well as between plasma metformin and arterial lactate [9].

Clinical presentations of metformin-associated lactic acidosis are non-specific. Severe hypotension with reduced systemic vascular resistance and respiratory failure requiring mechanical ventilation has also been reported [9,10].

An aggressive treatment strategy for metformin-associated lactic acidosis is recommended. Treatment for metformin-associated lactic acidosis includes adequate supportive care, management of concurrent disease, correction of acidemia, acceleration of lactate metabolism, and elimination of the offending drug by renal excretion or dialysis [11].

In our patients the early and aggressive treatment with haemodialfiltration could have improved the outcome even in the presence of very severe acidosis. Metformin is a small, non-plasma protein-bound molecule that was dialyzed out of the body and subsequent full correction of acid-base status and lactacidemia after 12 hours of continuous venovenous hemodiafiltration was observed in both patients.

Dialysis techniques are the adequate treatment of lactic acidosis in diabetic patients treated with metformin since it rapidly corrects the acid-base disorders and partially removes the metformin [12].

Since metformin-associated lactic acidosis is associated with a mortality rate as high as 50%, attention should be focused on prevention through awareness of the risk factors [13]. Identification and recognition of cautions and contraindications are the keys to reducing morbidity and mortality.

## Conclusion

Metformin-associated lactic acidosis is a rare, preventable, but life-threatening adverse event and should be strongly suspected in patients presenting with high-anion gap metabolic acidosis and high blood lactate concentration [11,14]. The daily metformin dosage should be no more than 2.5 g and the dosage o fmetformin should be reassessed as the patient ages [15].

## Competing interests

The authors declare that they have no competing interests and confirm that all authors have seen and agree with the contents of the manuscript and agree that the work has not been submitted or published elsewhere in whole or in part.

## Authors' contributions

JS conducted the literature review and carried out the review of the patient's medical record. PP participated in the preparation of the manuscript and revised the article for intellectual content details. All authors read and approved the final manuscript.

## Consent

Written informed patient consent was obtained for publication
